# Challenging Diagnostic Process for a Malignant Peritoneal Mesothelioma Patient With Ascites and Pleural Effusion: A Case Report and Review of the Literature

**DOI:** 10.3389/fonc.2022.784064

**Published:** 2022-03-15

**Authors:** Xiaofeng Zeng, Ke Xu, Liying Zhang, Xiaoli Huang

**Affiliations:** ^1^ The Center of Gerontology and Geriatrics, National Clinical Research Center for Geriatrics, West China Hospital, Sichuan University, Chengdu, China; ^2^ Sichuan Cancer Hospital & Institute, Sichuan Cancer Center, School of Medicine, University of Electronic Science and Technology of China, Chengdu, China

**Keywords:** ascites, pleural effusion, malignant peritoneal mesothelioma, diagnosis, case report, immunohistochemistry

## Abstract

Malignant peritoneal mesothelioma (MPM) is a sporadic and fatal disease of the peritoneal lining. Its diagnosis has been known to be challenging, time-consuming, and money-consuming. In this paper, we report an MPM case of a 58-year-old man with severe abdominal distension. After he had received all kinds of auxiliary examination, including computed tomography scans of the chest and whole abdomen, examinations of peripheral and pleural fluid, positron emission tomography, and twice fine-needle peritoneal biopsies, his disease still could not be confirmed. Eventually, the patient was diagnosed with MPM through laparoscopic biopsy and IHC. From this case, we concluded that clinicians can gradually discover and diagnose the disease through 1) high platelet and CA125 levels and CT imaging results, 2) cytologic examinations of ascites and pleural fluid, 3) peritoneal biopsies (fine-needle biopsy, laparoscopy biopsy), and 4) histopathological examinations and immunohistochemistry findings. The diagnostic process involving this patient can be an example to demonstrate the effectiveness of various auxiliary examination methods in MPM diagnosis.

## Introduction

Malignant peritoneal mesothelioma (MPM) is a rare and fatal disease of the peritoneal lining (1). When mentioning the cause, asbestos exposure remains the most definite risk factor for MPM, although it has been an infrequent factor, and only 8% of victims declared previous exposure (2). Studies have found that deletion of the BRCA-associated protein 1 (*BAP1*) gene, which participates in DNA repair and apoptosis, is related to the occurrence of MPM (3, 4). Therefore, young patients with MPM or patients with a family history of MPM should be tested for the presence of *BAP1* gene mutations.

On one hand, due to the low incidence of MPM, there are fewer reports about MPM. Unlike pleural mesothelioma, which accounts for most mesotheliomas, MPM only represents 7%–10% of cases (1, 5). It has been reported that there are approximately 300 new cases each year in the United States (5). On the other hand, the clinical presentations of MPM are overwhelmingly atypical, always showing non-specific signs and symptoms, such as abdominal pain, bloating, and massive ascites (6). Since there is no specific test to distinguish MPM from other possible maladies, it remains a challenge for physicians and healthcare providers to confirm the diagnosis. For the above reasons, we believe that the report on the diagnosis process of MPM is meaningful. Here, we present an efficient flow of subsequent tests which could be utilized to diagnose MPM when clinicians always received meaningless results.

This case report describes the detailed diagnostic process involving a 58-year-old man with significant ascites. We truly experienced the difficulty of MPM diagnosis and would like to demonstrate the effectiveness of various auxiliary examination methods for diagnosing this case.

## Case Report

In August 2020, a 58-year-old man was hospitalized in the Center of Gerontology and Geriatrics, West China Hospital, Sichuan University due to chest tightness. The patient’s abdominal CT only showed a small amount of pelvic effusion, and all of the tumor markers were at a normal level (**Table 1**). He underwent gastrointestinal endoscopy during the hospital stay, and the gastroscopy results were unremarkable. Colonoscopy showed a wide basal polyp of 0.3 cm in diameter in the colon and three flat polyps in the rectum (0.2, 0.2, and 0.3 cm in diameter), and all polyps were removed successfully. On pathological examination, the excised polyps proved to be hyperplastic. He was diagnosed with paroxysmal atrial fibrillation, hypertension, gallbladder stones, and prostate calcification before discharge.

**Table 1:** Laboratory results for serum, ascites, and pleural fluid.

	CA125 (U/ml)	CA15-3 (U/ml)	Cytokeratin 19 fragment (ng/ml)	CEA (ng/ml)	Tubercular antibody	ADA (IU/ml)
Normal state^a^ in the blood	<24	<24	<3	<5	Negative	–
Serum (the first hospitalization)	13.3	6.98	4.98	1.32	–	–
Serum	232.00	49.10	11.40	1.18	–	–
Ascites	734.00	83.90	84.2	0.54	Positive	37.8
Pleural fluid	1,079.00	63.60	21.70	0.44	Positive	16.2

–, not available.

a Normal state in West China Hospital.

Four months later, the patient was readmitted to our department with severe abdominal distension for the past 2 months. The only additional symptom the patient had was poor appetite and reduced food intake. No person in the patient’s family had records of a similar disease. Physical examination yielded the following characteristics: height, 174 cm; weight, 105 kg; body mass index, 34.68 kg/m^2^; and abdominal circumference, 127 cm. On abdominal examination (insufficient palpation), we found abdominal distension, negative peritoneal irritation signs, positive mobile dullness, and normal bowel sounds. There was nothing special noted in the lung and heart examinations.

**Figure 1:**
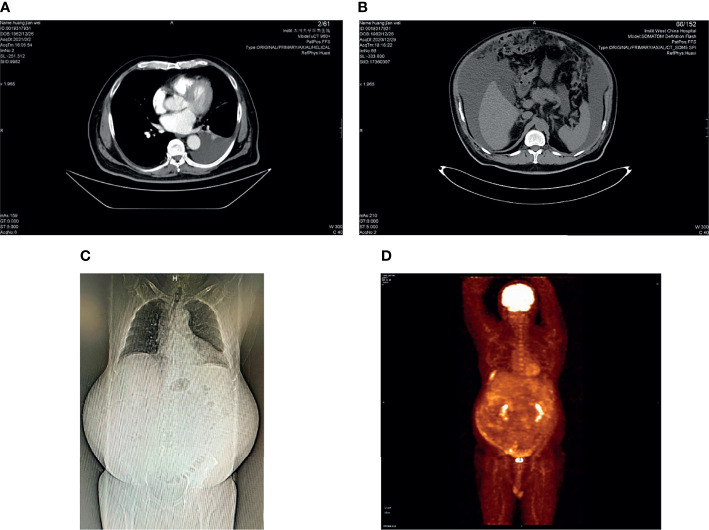
CT and PET-CT photos: **(A)** left pleural effusion, axial CT scan; **(B)** massive ascites, axial CT scan; **(C)** coronal CT scan; **(D)** PET-CT: there was inhomogeneous peritoneal thickening accompanied by abnormally increased glucose metabolism. We were unsure if this was a tumor or just inflammation.

Abdominal computed tomography (CT) demonstrated a large amount of effusion in the abdomen and pelvis, along with a small amount of pleural effusion (**Figures 1** **A–C**). Enhanced CT of the entire abdomen depicted a thickened peritoneum and omentum in addition to massive ascites and pelvic fluid. Laboratory test results showed that platelets had increased significantly (552 × 10^9^/L), total protein (55.1 g/L) and albumin (32.7 g/L) were slightly low, but there was normal liver and kidney function. Tumor markers CA125, CA15-3, and cytokeratin 19 fragment were abnormally high in the blood, pleural fluid, and ascites (**Table 1**). The patient was positive for tuberculosis antibodies in both the pleural fluid and ascites. Previous studies have shown that when pleural effusion adenosine deaminase (ADA) is ≥40 U/L, it has high sensitivity and specificity in identifying tuberculous pleurisy (7); however, the patient’s ADA status was not high enough to help diagnose tuberculosis (**Table 1**). Later, a series of tests to diagnose tuberculosis were all negative. Moreover, the patient was economically well off and lived a comfortable life. Repeated cytology examinations of the pleural fluid and ascites only found proliferative mesothelial cells.

After reviewing the history and results of laboratory and imaging tests, the patient could not be diagnosed with tuberculosis. Thus, we believed there was a high possibility that he had a malignant tumor. Positron emission tomography (PET)-CT was then conducted, and it showed inhomogeneous peritoneal thickening, which was accompanied by abnormally increased glucose metabolism. We were uncertain whether this was a tumor or just inflammation (**Figure 1D**). However, PET-CT revealed a small cyst in the right lobe of the liver that proved to be a tumor of the abdominal wall during the following examination.

The patient twice received a fine-needle peritoneal biopsy, and pathological examination of the sample found some abnormal cells. Immunohistochemistry (IHC) staining showed PCK (+), CK7 (+), CK5/6 (+), WT-1 (+), HBME-1 (+), and desmin (+), supporting mesothelial cell hyperplasia or mesothelial tumors. However, the reports from the pathological department said that the biopsy tissue was either too small to be fully confirmed or useless. Finally, laparoscopy was performed: there were turbid ascites and extensive adhesions in the abdominal cavity and many nodules (most likely tumor-implanted nodules) on the abdominal wall, omentum, organ surface, and between the organs. The abdominal viscera had adhered to a mass, and the suspected primary tumor could not be detected. The postoperative pathological report for the omentum and abdominal wall indicated malignant mesothelioma. Immunohistochemistry showed tumor cells WT-1 (+), CR (+), CK5/6 (+), HBME-1 individual (+), Des individual (+), CD31 (−), and CDX-2 (−), and the positive Ki-67 rate was approximately 30%, supporting the diagnosis of MPM.

**Figure 2:**
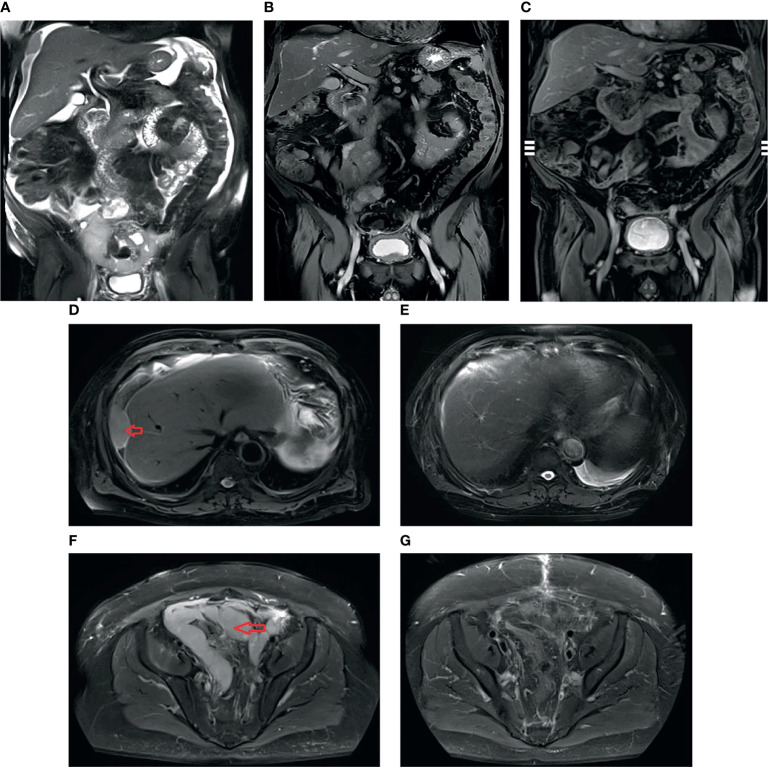
T2 sequence of coronal MRI: **(A)** before initial treatment with systemic chemotherapy (SC): massive ascites and the tumors; **(B)** after four cycles of SC: ascites disappeared, and the tumors became smaller and fewer in number; **(C)** after SC + cytoreductive surgery (CRS) + adjuvant chemotherapy treatment: the intra-abdominal tumor had resolved 3/4. T2 sequence of axial MRI: **(D)** before initial SC, arrow: peritoneal tumor; **(E)** after SC + CRS + adjuvant chemotherapy treatment, the tumor mass showed a complete response; **(F)** before initial SC, arrow shows “N”-shaped giant tumors; **(G)** after SC + CRS + adjuvant chemotherapy treatment, the tumors became smaller and fewer in number.

As his peritoneal cancer index (PCI) was >30 and he had extensive adhesions in the abdominal cavity, the patient was not immediately recommended to receive treatment with cytoreductive surgery (CRS) + hyperthermic intraperitoneal chemotherapy (HIPEC). Thus, he received four cycles of systemic chemotherapy (SC) with pemetrexed, cisplatin, and bevacizumab. After that, the tumor shrank significantly. He then underwent CRS and continued with two cycles of adjuvant chemotherapy. In more detail, each cycle of chemotherapy was used for 3 days, then 21 days apart; d0 represents the day before chemotherapy; d1, d2, and d3 represent the first day, second day, and third day of SC, respectively. The first and second SC cycles involved pemetrexed 900 mg d1 + cisplatin 50 mg d1–d2, 30 mg d3 + bevacizumab 400 mg d0; the third SC cycle and the twice postoperative SC cycles involved pemetrexed 700 mg d1 + cisplatin 40 mg d1, 30 mg d2–d3 + bevacizumab 400 mg d0 (the doses of pemetrexed and cisplatin decreased due to severe bone marrow suppression); the fourth SC cycle involved pemetrexed 700 mg d1 + cisplatin 40 mg d1, 30 mg d2–d3. Adverse reactions during chemotherapy included bone marrow suppression, immunodeficiency, and dysphagia, which improved after symptomatic treatment. We believed that the patient’s compliance was excellent, as he completed the established treatment plan on time and in quantity. The latest MRI showed that the intra-abdominal tumor had resolved by three-quarters of the original size, and ascites and pleural fluid were absent (**Figure 2**).

## Discussion

MPM is a hostile disease that has often spread over the entire abdomen and pelvis at diagnosis. In the current case, even though this patient was admitted to the second strongest hospital in China (8), the final diagnosis of MPM was only achieved after an extended clinical process and excessive costs. Thus, we believed it would be valuable to provide a report on his diagnosis process.

CT is preferred as the first-line imaging tool in diagnosing MPM (9), and CT signs include diffuse, irregular, or nodular thickening of the omentum and mesentery (10). However, some cases do not present these typical characteristics on CT imaging. A definite diagnosis of MPM requires histopathological examination of the peritoneal tissues, and the most reliable way to obtain biopsy samples is laparoscopy (11). MPM is classified into three histologic subtypes: epithelioid (75%–90% of cases), sarcomatoid, and biphasic subtypes (6). Moreover, IHC is needed to confirm the final diagnosis. The most sensitive IHC markers include calretinin (100%), Wilm’s tumor (WT-1, 94%), cytokeratin 5/6 (89%), and human mesothelial cell 1 (HBME-1) (2). It is generally agreed that at least two IHC markers should be positive to diagnose MPM (12). Immunohistochemistry demonstrated a loss of *BAP1* expression, which is reported in 55%–67% of instances (13), supporting the diagnosis of malignant mesothelial lesions. In addition, researchers have reported that platelets and CA125 can significantly increase in MPM patients (14, 15), and CA125 has particularly suggestive significance in the diagnosis and treatment of MPM (9). Many studies mentioned the ineffectiveness of PET-CT in MPM diagnosis (2), while PET-CT should be considered when a primary tumor site cannot be located after various other examination methods. Sole reliance on the cytologic examination of ascites and pleural effusion makes it challenging to diagnose MPM, as the fluid contains no or few abnormal cells in the great majority of cases (9).

Treatment with CRS plus hyperthermic intraperitoneal chemotherapy (HIPEC) is the standard course for MPM, and the epithelioid subtype is the best candidate for CRS plus HIPEC (16). For inoperable or non-resectable patients, which are not suitable for the standard treatment, the guidelines recommend choosing SC treatment rather than palliative care (9). A recent study showed that better survival was independently associated with both the treatment combination of CRS plus HIPEC and surgery plus SC (17), although the overall survival of patients treated with SC plus CRS was approximately 1 year (18). If MPM patients cannot hold a beneficial prognostic factor, the treatment mode of CRS + HIPEC + adjuvant SC should be adopted (9). The beneficial prognostic factors include female sex, non-elderly (<60 years), epithelioid subtype, no lymph node metastasis, and Ki-67 ≤9% (19). The study of Kusamura et al. found that the proliferation marker Ki-67 was an independent determinant of survival (20). Programmed cell death ligand-1 (PD-L1) is another prognostic marker, and when expressed by epithelioid MPM patients, it is indicative of better overall and progression-free survival (21). For the chemotherapy regimen, pemetrexed in combination with cisplatin is the most recommended regimen choice (9). Studies have found that vascular endothelial growth factor (VEGF) is an essential mediator of malignant ascites and pleural effusion formation; therefore, anti-VEGF bevacizumab could effectively suppress the formation of malignant ascites and pleural effusion (22).

There are some limitations to our report. First, we did not indicate the MPM histological pattern of the patient, although it is vital for predicting prognosis. The pathology department provided us with a report that did not specify the histological type; thus, we have not provided the patient’s pathological MPM subtype in this report. Second, the patient underwent many auxiliary examinations to diagnose MPM. It was an unpleasant experience and a considerable expenditure for the man and his family. However, the significance of the case cannot be ignored. First of all, there are few case reports focusing on MPM. The diagnostic process of the case was challenging, and the report is educational. Because we repeatedly obtained uncertain evidence that could not be used to diagnose MPM, the patient went through nearly all the recommended methods. Thus, we believe this case is a valuable example of the effectiveness of various auxiliary examination methods in MPM diagnosis. Lastly, this case indicates that investigations into the treatment modality of MPM are promising for providing better survival. In this case, the patient’s prognosis was surprisingly excellent after receiving SC + CRS + adjuvant chemotherapy treatment.

From this case, we learned that MPM can be gradually discovered and diagnosed through 1) laboratory examinations of blood and ascites, such as high platelet and CA125 levels, and imaging tests, such as CT imaging results, to provide information on an abnormal peritoneum; 2) cytologic examination to find unusual cells; 3) peritoneal biopsy, including fine-needle biopsy and laparoscopy biopsy, to retrieve peritoneal tissues; and 4) histopathological examination and IHC findings to confirm the final diagnosis. Moreover, we noticed that the patient achieved a fairly satisfactory survival outcome, even though MPM is a disease with a poor prognosis. Thus, we believe that research focusing on the treatment of MPM holds further promise in the future.

## Data Availability Statement

The original contributions presented in the study are included in the article/**Supplementary Material**. Further inquiries can be directed to the corresponding author.

## Ethics Statement

Written informed consent was obtained from the individual(s) for the publication of any potentially identifiable images or data included in this article.

## Author Contributions

XZ contributed to the conception and design, data acquisition and analysis, literature review, article drafting, and manuscript revision. KX did much work acquiring and interpreting data, as she was the patient’s attending physician, and she also took part in manuscript drafting and revision. LZ contributed to the conception and design and literature review and took part in drafting the article. XH guided the whole process, prepared the report, and revised the paper critically and crucially. All authors gave final approval of the version to be published.

## Funding

This case report was funded by the Health and Scientific Research for Cadres in Sichuan Province (Grant no. 2020-103 and 2019-104).

## Conflict of Interest

The authors declare that the research was conducted in the absence of any commercial or financial relationships that could be construed as a potential conflict of interest.

## Publisher’s Note

All claims expressed in this article are solely those of the authors and do not necessarily represent those of their affiliated organizations, or those of the publisher, the editors and the reviewers. Any product that may be evaluated in this article, or claim that may be made by its manufacturer, is not guaranteed or endorsed by the publisher.
