# Supplementary material for: Challenging Diagnostic Process for a Malignant Peritoneal Mesothelioma Patient With Ascites and Pleural Effusion: A Case Report and Review of the Literature
Source: Front Oncol. 2022 Mar 15;12:784064. doi: 10.3389/fonc.2022.784064 (PMC8964484; doi:10.3389/fonc.2022.784064)
Supplement: Supplementary file 1 [file DataSheet_1.pdf]

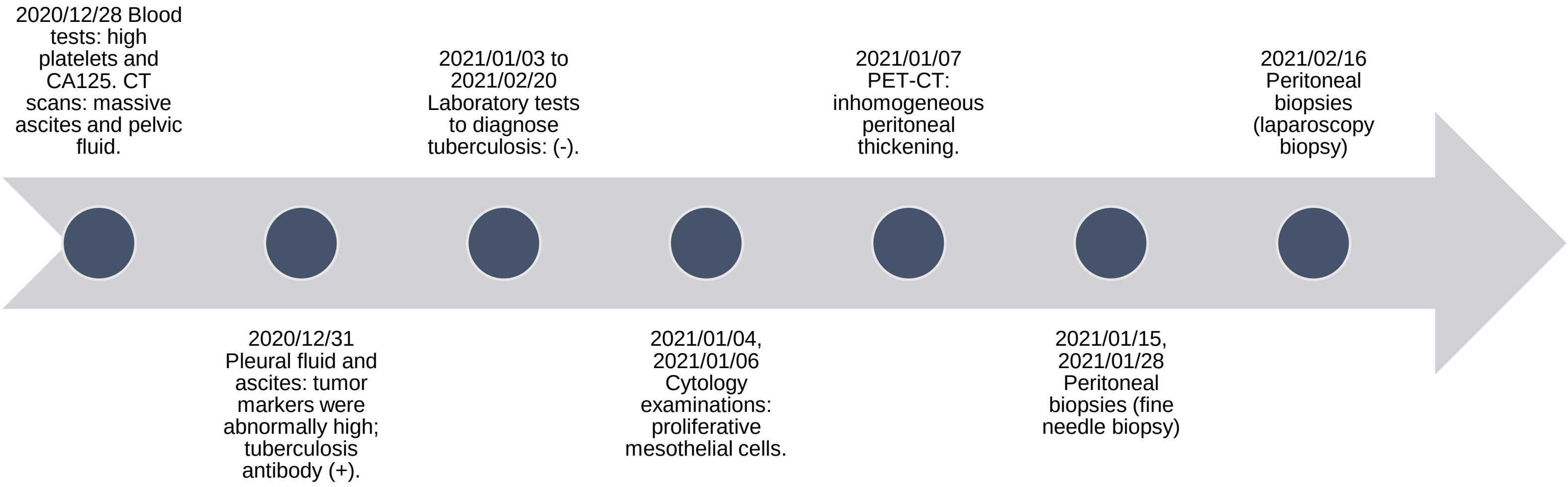

Supplement Figure. 1 Timeline of critical findings of diagnostic information.

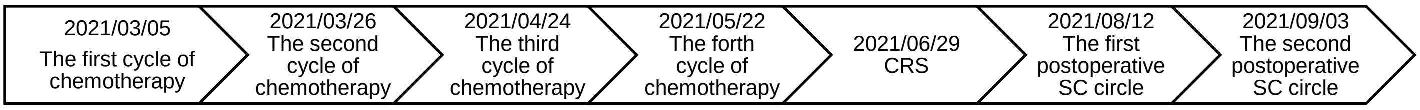

Supplement Figure. 2 Timeline of the main treatments of the patient.
